# Real-World Feasibility, Toxicity, and Early Outcomes of Neoadjuvant Pembrolizumab-Based Systemic Therapy in Early Triple-Negative Breast Cancer: A Single-Centre Chinese Cohort from Hong Kong

**DOI:** 10.3390/cancers18203339

**Published:** 2026-10-16

**Authors:** Yuk-Fong Grace Lo, Kuen Chan, Sung Inda Soong

**Affiliations:** Department of Clinical Oncology, Pamela Youde Nethersole Eastern Hospital, Hong Kong SAR, China

**Keywords:** triple-negative breast cancer, neoadjuvant chemotherapy, pembrolizumab, KEYNOTE522, pathological complete response, Chinese, Hong Kong, real-world

## Abstract

Background: Neoadjuvant treatment for early-stage triple-negative breast cancer has been widely used, and the KEYNOTE-522 (KN522) regimen has become a standard of care for early-stage TNBC, with alternative treatment options including a dose-dense anthracycline/taxane-based regimen for patients without access to neoadjuvant immunotherapy. Nevertheless, real-world evidence in Chinese populations, especially high-risk cohorts such as older populations and those with substantial nodal burden, is limited regarding both efficacy and tolerability. This study describes the outcomes associated with neoadjuvant systemic therapy in a Hong Kong Chinese cohort. Objectives: This study aimed to describe the feasibility, treatment delivery, tolerability and safety of the KN522 regimen in stage I-III TNBC in a Chinese cohort in Hong Kong. Another cohort treated with dose-dense neoadjuvant chemotherapy, which is also a standard-of-care treatment alternative, is presented to provide context of local treatment practice. Methods: We conducted a retrospective observational cohort study of 59 patients with stage I-III TNBC who received neoadjuvant systemic treatment prior to breast surgery at a tertiary centre in Hong Kong between February 2022 and June 2025. The primary cohort received the KN522 regimen (*n = 30*). A contextual reference cohort received dose-dense epirubicin/cyclophosphamide followed by paclitaxel (*n = 29*) to describe local treatment patterns. Descriptive outcomes included pathological complete response (pCR), treatment delivery, adverse events, surgery completion, and exploratory event-free survival (EFS). Analyses were primarily descriptive; between-cohort comparisons were exploratory in nature. Results: Fifty-nine patients were included overall: 30 treated with the KN522 regimen and 29 treated with dose-dense neoadjuvant chemotherapy. In the KN522 cohort, the median age was 56 years, 80.0% of patients were postmenopausal, and 33.3% had N3 disease. Pathological complete response occurred in 13 of 30 patients (43.3%; exact 95% CI 25.5–62.6%). Grade ≥3 chemotherapy-related adverse events occurred in 15 patients (50.0%), immune-related adverse events occurred in 11 patients (36.6%), and Grade 3 or higher immune-related adverse events occurred in four patients (13.3%). Pembrolizumab was suspended in six patients (20.0%), while all 30 patients proceeded to definitive surgery. In the dose-dense chemotherapy cohort, the pCR rate was 31.0%, with a grade ≥3 chemotherapy-related adverse event rate of 20.7%, and all patients proceeded to definitive surgery. Conclusions: In this descriptive real-world single-centre Hong Kong study, the neoadjuvant KN522 regimen was feasible in Chinese patients with early TNBC, although treatment-related toxicity and treatment modification were common. The observed pCR rate was lower than that reported in the original KN522 trial, possibly reflecting differences in patient selection and real-world treatment delivery patterns. Larger multi-centre studies are needed to evaluate outcomes in Chinese and other Asian populations.

## Simple Summary

Neoadjuvant pembrolizumab combined with chemotherapy is widely used for high-risk early triple-negative breast cancer (TNBC). However, real-world feasibility and safety evidence from Chinese and other Asian populations remains limited. We retrospectively describe the feasibility, toxicity and safety of the KEYNOTE-522 regimen (KN522) in 30 patients and a dose-dense chemotherapy cohort in 29 patients as a contextual reference cohort with early TNBC treatment in a tertiary hospital in Hong Kong.

## 1. Introduction

Breast cancer is the most common cancer in women globally [1] and the second-most common cancer in Hong Kong [2]. TNBC is a special entity that accounts for approximately 15% of breast cancers, with higher incidence rates in Afro-Caribbean and Asian populations [3,4]. It is associated with the germline Breast Cancer Gene (BRCA) and could be hereditary in nature. Triple-negative breast cancer (TNBC) has a more aggressive disease tempo associated with increased mortality among women [5].

Neoadjuvant systemic treatments, including induction dose-dense anthracycline/taxane-based chemotherapy and the introduction of phase III KN522 trials, shed light on this group of patients, showing promising event-free survival [6], pathological complete response rate and improvement translated to overall survival [7,8]. Recently published final analysis showed clinically meaningful benefit in progression-free survival and overall survival with the use of neoadjuvant immunotherapy after a median follow-up period of 7.8 years [9], which further supports the application of pembrolizumab in a neoadjuvant setting.

Nevertheless, the combination of immunotherapy with chemotherapy can have side effects on patients in routine clinical practice. Specific efficacy and side effect profiles have not been well studied in a Southeast Asian Chinese population [10]. A proportion of patients fail to complete the course of neoadjuvant treatment due to side effect profiles. Higher rates of dose reduction have been reported due to the extended use of the KN522 regimen in frailer patients, such as with Eastern Cooperative Oncology Group (ECOG) performance status (PS) 2 patients and older patients [11,12,13,14]. These groups were excluded in the original trial design. The expansion of selection criteria to N3 disease is also frequently encountered in real life, which was not included in the KN 522 trial.

In this retrospective descriptive observational study, we included patients diagnosed with early stage I-III disease who received neoadjuvant systemic treatment, during the period of February 2022 to June 2025, and who were treated in Pamela Youde Nethersole Eastern Hospital in Hong Kong. This real-world study aims to describe the feasibility, treatment delivery and toxicity profile of the KN522 regimen in a Chinese cohort, especially those with substantial nodal burden, in Hong Kong. A dose-dense chemotherapy cohort, which is an alternative standard of care regimen, is presented to provide context for local treatment variation, and all between-cohort analyses were exploratory and hypothesis-generating.

## 2. Materials and Methods

### 2.1. Participants

A total of 59 patients diagnosed with non-metastatic, histologically confirmed triple-negative breast cancer (Oestrogen Receptor score 0–2, Progesterone Receptor score 0–2, Allred score 0–2 on histological analysis) and HER-2 negative (HER-2 IHC 0/1 or HER-2 IHC ≥ 2 with ISH/DISH/FISH negative) stage I-III TNBC during the period of February 2022 to June 2025 in Pamela Youde Nethersole Eastern Hospital in Hong Kong were enrolled. Ethical approvals were obtained from the Central Institutional Review Board of Hospital Authority, Hong Kong (Ref No. CIRB-2026-137-5).

### 2.2. Neoadjuvant and Adjuvant Treatments

Both the neoadjuvant KN522 regimen and dose-dense chemotherapy are standard-of-care regimens for early TNBC in our institution. Treatment selection was determined by multi-disciplinary discussion, patient preference and access to immunotherapy. We describe real-world outcomes in two consecutive cohorts receiving these standard regimens.

Patients who received neoadjuvant pembrolizumab + chemotherapy and induction dose-dense chemotherapy during the period were included, comprising 30 patients in the KN522 cohort and 29 patients in the dose-dense chemotherapy cohort.

Patients in the KN522 cohort received pembrolizumab every 3 weeks with weekly paclitaxel and carboplatin for 12 weeks, followed by pembrolizumab with epirubicin and cyclophosphamide every three weeks for four cycles.

Patients in the dose-dense neoadjuvant chemotherapy cohort received dose-dense epirubicin + cyclophosphamide every two weeks for four cycles, followed by paclitaxel every 2 weeks for four cycles, for a total of eight cycles.

Treatment continued until withdrawal of treatment due to an adverse event or until completion of neoadjuvant treatment followed by definitive surgery if suitable. Adjuvant systemic treatment (adjuvant pembrolizumab or adjuvant capecitabine as per CreateX trial [15] or to clinical trial in other centres as per patient’s desire) and adjuvant radiotherapy were offered depending on the ypTNM staging.

### 2.3. Endpoint

This is a descriptive retrospective observational study. The primary focus was the characterisation of the treatment delivery pattern, toxicity, pathological response, surgery completion rate and exploratory EFS among patients receiving the KN522 regimen. The dose-dense chemotherapy cohort was included as a reference group reflecting local treatment practice, usually available for those with financial difficulty for immunotherapy. The study was not designed or powered to estimate causal effect of pembrolizumab or to establish comparative effectiveness between treatment regimens.

The primary descriptive outcome was pathological complete response rate (pCR). Secondary outcomes included adverse event profile, immune-related adverse events, treatment modification, treatment discontinuation rate because of toxicity, and completion of definitive surgery. Exploratory Event-Free Survival (EFS) was evaluated as an exploratory time-to-event outcome. pCR was defined as no residual invasive disease in the breast and sampled regional lymph nodes at definitive surgery (ypT0/Tis ypN0), based on local pathology reports. Risk factors such as age at diagnosis, menopausal status, staging according to the TNM staging system created by the American Joint Committee on Cancer, dose modification percentage, treatment response, adjuvant treatment received, and adverse event or immune-related adverse event and its grading as classified by Common Terminology Criteria for Adverse Events (CTCAE) were recorded from retrospective clinical records for analysis. Between-cohort differences in pCR, toxicity and EFS were examined descriptively and were not prespecified as a primary hypothesis.

### 2.4. Statistical Analysis

Analyses were performed using R version 4.5.3 and IBM SPSS Statistics version 31. Analyses were primarily descriptive and focused on the KN522 cohort. The dose-dense chemotherapy cohort was included to provide context regarding local treatment practice variation. Continuous variables were summarised using mean and standard deviation and, where appropriate, median and interquartile range. Categorical variables were summarised using frequencies and percentages. Between-cohort comparisons used Welch’s independent samples t-test for age, the Mann–Whitney U test for skewed continuous treatment delivery variables, Pearson chi-square tests for categorical variables when expected cell counts were adequate, and Fisher’s exact tests for sparse 2 × 2 comparisons. Fisher’s exact test was used as the primary test for the main pCR comparison because of the small sample size. These comparisons were exploratory and not adjusted for multiple testing.

The primary descriptive outcome was pathological complete response. pCR rates were summarised by treatment cohort using *n*/*N (%)* and exact binomial 95% confidence intervals, and between-cohort differences were presented using absolute risk difference and odds ratios with 95% confidence intervals. External benchmark pCR rates from KN522 and an ESMO real-world cohort were included for descriptive context only.

Exploratory logistic regression was used to examine associations of pCR and of treatment delivery outcome. Univariable models were fitted first for treatment cohort and selected baseline clinical variables. Because the number of pCR events was limited, multivariable models were kept parsimonious and included the treatment cohort plus one additional baseline factor at a time. These analyses were considered hypothesis-generating.

Exploratory event-free survival was analysed as a time-to-event outcome, measured from treatment start to the first EFS event. Patients without an event were censored at the administrative data cut-off date of 12 July 2026. Survival was estimated using the Kaplan–Meier method, and median follow-up was estimated using the reverse Kaplan–Meier method. Because only ten EFS events occurred, EFS analyses were considered exploratory and hypothesis-generating outcomes. An unadjusted log-rank test and an unadjusted Cox proportional hazards model were used for exploratory between-cohort summaries and are reported for transparency only.

This study follows the STROBE checklist to ensure clear and accurate reporting of the observational data.

## 3. Results

### 3.1. Participant Characteristics

A total of 59 patients with early triple-negative breast cancer were included, with 30 in the KN522 cohort and 29 in the chemotherapy cohort. Baseline characteristics are presented in Table 1. The median age was 56 years (IQR 51–64 years) overall. In the KN522 cohort, the median age was 56 years and 24 of 30 patients (80%) were postmenopausal. Ten patients (33.3%) had N3 disease. Stage II disease was most common (56.7%), followed by stage III (36.7%) and stage I disease (6.7%). In the dose-dense chemotherapy cohort, the median age was 57 years and 20 of 29 patients (69%) were postmenopausal. Six patients (20.7%) had N3 disease. Stage II disease was most common (58.6%), followed by stage III (37.9%) and stage I disease (3.4%). These descriptive differences highlight the heterogeneity of treatment selection in routine clinical practice.

### 3.2. Pathological Complete Response

Pathological complete response (pCR) was summarised by treatment cohort, as shown in Table 2.

**Figure 1:**
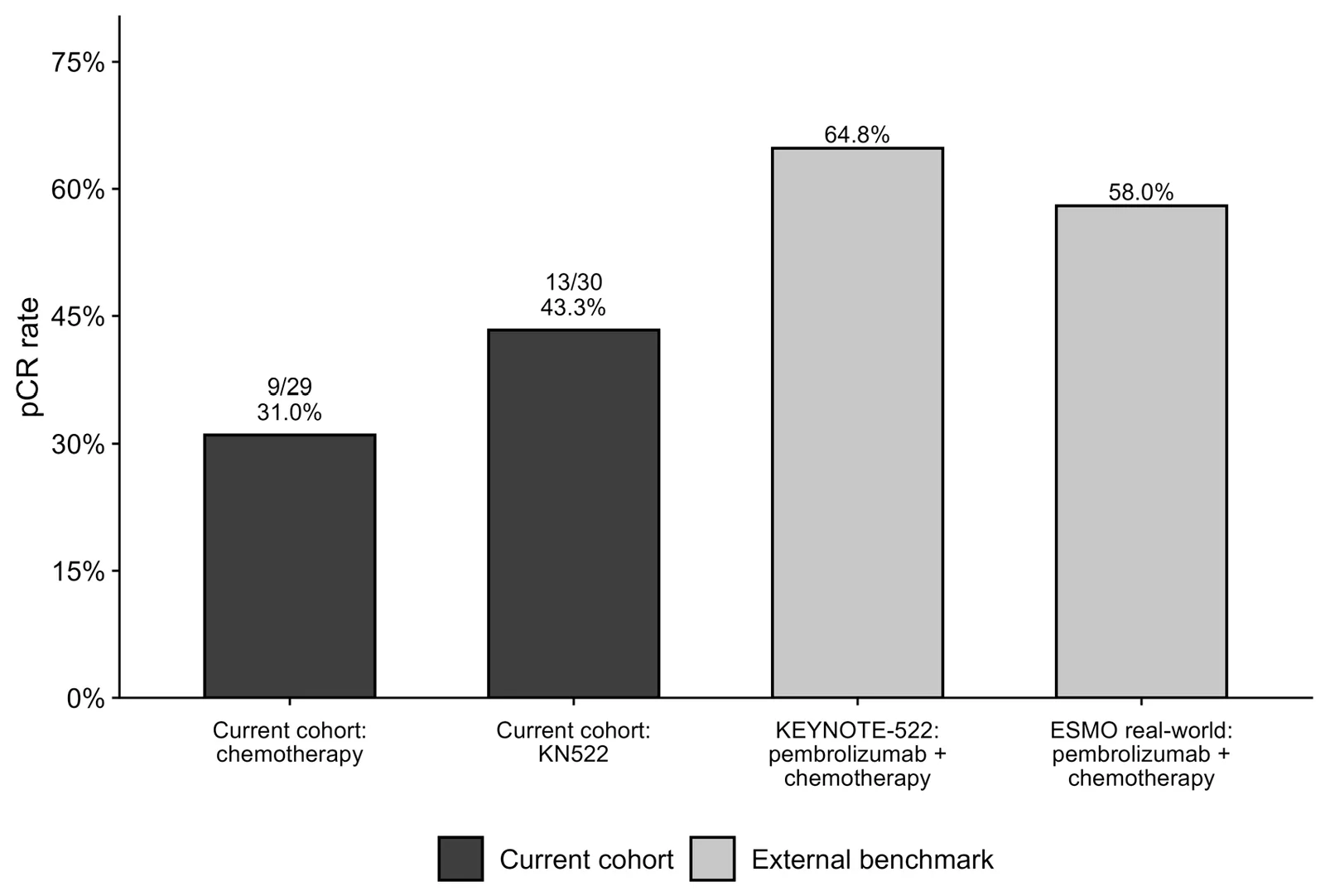
Observed pathological complete response rate by cohorts with external benchmarks. Note. pCR = pathological complete response. External benchmark bars for KEYNOTE-522 and the ESMO real-world cohort are provided for descriptive context only and were not included in inferential testing.

Within the KN522 cohort, pCR was observed in 13 of 30 patients, and the observed pCR rate in the KN522 cohort (43.3%; exact 95% CI 25.5–62.6%) was below the published rates of 64.8% in KN522 and 58.0% in the ESMO real-world cohort. These external rates are shown in Table 2 and Figure 1 for context only and were not statistically compared with the current cohort. In the dose-dense chemotherapy cohort, pCR was observed in nine of 29 patients (31.0%; exact 95% CI 15.3–50.8%). These rates are presented primarily as cohort-specific descriptive observations.

Further exploratory between cohort review was performed in Supplementary Table S1.

### 3.3. Safety and Treatment Modification

Chemotherapy-related adverse events and exploratory cross-cohort analyses are reported in Table 3 and summarised in Figure S1**.** Any grade of chemotherapy-related adverse event was recorded in 96.7% of the KN522 cohort and 86.2% of the chemotherapy cohort, with no statistically significant difference in the exploratory between-cohort comparison (*p* = 0.195). Grade 3 or higher adverse events were recorded in 15 of 30 patients (50.0%) in the KN522-based cohort and six of 29 patients (20.7%) in the dose-dense chemotherapy cohort.

In the KN522-based cohort, peripheral neuropathy was recorded in four of 30 patients (13.3%), constipation in one patient (3.3%), and any-grade neutrophil count decrease in 12 patients (40.0%). In the dose-dense chemotherapy cohort, peripheral neuropathy was recorded in 17 of 29 patients (58.6%), constipation in eight patients (27.6%), and any-grade neutrophil count decrease in four patients (13.8%). Grade 3 or higher neutrophil count decrease was recorded in seven patients (23.3%) in the KN522-based cohort and three patients (10.3%) in the chemotherapy cohort. Anaemia, nausea, fatigue, and rash were recorded in both cohorts at varying frequencies.

Immune-related adverse events were recorded in 36.6% of the KN522 cohort. Type- and grade-specific details are presented in Table S2 and Figure S2. Hypothyroidism was recorded in three patients (10.0%). Hepatitis was also recorded in three patients (10.0%). Adrenal insufficiency, hyperthyroidism, pneumonitis, and severe skin reactions were each recorded in a small number of patients.

All three recorded hepatitis events were Grade 3 or higher, comprising two Grade 3 events and one Grade 4 event. A Grade 3 or higher severe skin reaction was also recorded in one patient. Pembrolizumab suspension occurred in 20.0% of the KN522 cohort, and an immune-related intervention was recorded in 33.3%, both of which were specific to patients receiving the KN522 treatment strategy.

Treatment delivery outcomes and exploratory cross-cohort comparisons for treatment delivery outcomes are summarised in Table 3. In the KN522 cohort, 18 of 30 patients (60%) underwent at least one chemotherapy dose modification, and the median lowest delivered chemotherapy dose was 80%. No patient discontinued neoadjuvant treatment and proceeded directly to upfront surgery. All 30 patients completed definitive surgery, and 24 patients (80%) received postoperative radiotherapy.

In the dose-dense chemotherapy cohort, 16 of 29 patients (55.2%) underwent a dose modification; the median lowest delivered dose was 90% and two patients (6.9%) discontinued neoadjuvant treatment and proceeded to upfront surgery. All 29 patients completed definitive surgery, and 22 patients (75.9%) received postoperative radiotherapy.

As for postoperative adjuvant treatment choice, in the KN522 cohort, 23 of 30 patients (76.7%) received adjuvant pembrolizumab; four patients (13.3%) received capecitabine. In the chemotherapy cohort, 19 of 29 patients (65.5%) received capecitabine.

### 3.4. Exploratory Associations with Pathological Complete Response

Exploratory logistic regression models were used to describe any associations between selected clinical variables and pCR. Separate univariable logistic regression models were fitted for treatment cohort and selected baseline clinical characteristics. Exploratory adjusted models were then fitted by adding one clinically relevant baseline factor to each treatment cohort at a time. This parsimonious approach was used because of the limited sample size and number of pCR events; results are presented in Table S3. Logistic regression Wald tests were applied.

These models should be interpreted as descriptive associations within this retrospective cohort and not as adjusted estimates of comparative treatment efficacy.

In the univariable model, the exploratory odds ratio for pCR in the KN522 cohort relative to the dose-dense chemotherapy cohort was 1.70 (95% CI, 0.58 to 4.94, Wald *p* = 0.330). In exploratory models that additionally included overall clinical stage or node-positive disease, the corresponding odds ratios were 1.74 (95% CI, 0.59 to 5.17, *p* = 0.317) and 1.60 (95% CI, 0.54 to 4.72, *p* = 0.395), respectively. These estimates are presented as hypothesis-generating observations.

No baseline clinical factor demonstrated a statistically reliable association with pCR in these exploratory analyses. Increasing age showed an exploratory odds ratio of 0.55 per 10-year increase (95% CI, 0.30 to 1.01, *p* = 0.052), while the presence of any comorbidity also showed an exploratory odds ratio of 0.36 (95% CI, 0.11 to 1.11, *p* = 0.075).

### 3.5. Exploratory Event-Free Survival

Exploratory event-free survival was summarised descriptively in each cohort. EFS was calculated from treatment initiation to first prespecified EFS event, including local recurrence, distant progression or death, or censoring. Results are presented in Table 4. The data cut-off date was 12 July 2026. Ten EFS events occurred overall among 59 patients (16.9%), including seven of 30 patients in the KN522 cohort (23.3%) and three of 29 patients in the chemotherapy cohort (10.3%). Among patients who had an event, the median time to that event was 23.2 months in the KN522 cohort and 15.2 months in the chemotherapy cohort. Distant recurrence was recorded in nine out of the ten patients. Five deaths were recorded during follow-up.

Cohort-specific Kaplan–Meier estimates are shown in Table 5. Median follow-up was 28.2 months overall, and median EFS was not reached in either cohort. The estimated 12-month EFS was 96.7% in the KN522 cohort and 96.6% in the chemotherapy cohort. By 24 months, the EFS estimates were 80.6% in the KN522 cohort and 88.3% in the chemotherapy cohort. A further exploratory between-cohort review was performed (see Supplementary Table S4) and presented as hypothesis-generating observations.

The observed EFS patterns reflect different real-world perioperative pathways in a clinical setting rather than an isolated effect of pembrolizumab. The cohorts differed in treatment selection, baseline risk (including the proportion of N3 disease), chemotherapy backbone and postoperative treatment.

Figure S3 shows the Kaplan–Meier curves with their confidence bands and the number of patients remaining at risk. The number at risk also fell to single figures in both cohorts beyond 30 months, and more steeply in the KN522 cohort, so the later estimates rest on very few patients.

## 4. Discussion

The treatment landscape of early-stage TNBC has shifted dramatically since the introduction of neoadjuvant systemic treatment [16,17,18].

Asian patients are usually underrepresented in clinical trials. In the global KEYNOTE-522 trial of 1174 randomised patients, 216 patients were enrolled from Asia (~18.4%) and 50 Chinese patients from Taiwan were enrolled (4.3%) [19]. Patients from Mainland China and the Greater Bay area were not enrolled in the global trial [19].

Our real-world data provide an insight from a group of Chinese patients who had the same regimen in clinical practice in Hong Kong. Expert consensus recommendations have been published in Hong Kong to provide guidance on the suitability of the KEYNOTE-522 regimen in early TNBC patients [20]. To our knowledge, this is among the first real-world studies to evaluate the KN522 regimen in a Chinese cohort with a heavy burden of N3 disease.

In our study, 30 patients with early-stage TNBC were treated with a KEYNOTE-522 regimen. In this cohort, pCR was observed in 13 of 30 patients (43.3%), Grade 3 or higher adverse events were observed in 15 patients (50%), and all patients subsequently proceeded to definitive surgery. The dose-dense chemotherapy cohort also demonstrated pCR of 31%, and Grade 3 or higher adverse events were observed in 20.7% of patients; all patients proceeded to definitive surgery. Both cohorts delivered a lower pCR rate compared with the original landmark trial, where the KN522 cohort achieved a 64.8% pCR rate and the chemotherapy cohort achieved 51.2% [21]. Such a cross-study review is descriptive only because the populations differed in age, nodal burden, treatment regimen differences and clinical setting. Although the observed cohort-specific numbers differ, the small sample size and wide confidence interval limit direct comparison between cohorts, and therefore the exploratory odds ratio and *p*-value should be regarded as hypothesis-generating descriptions of associations within this retrospective cohort.

Several clinical and demographic imbalances may have contributed to the demonstrated pCR rate and EFS.

In the KEYNOTE-522 landmark trial, N3 disease was excluded in the trial design [21]. Only two out of 1174 patients in the global KEYNOTE-522 trial in 2023 had N3 disease (0.2%) [19]. Our real-world study included groups of higher risk patients, such as older patients and those with higher nodal burden, where 33.3% in the pembrolizumab cohort (10/30) and 20.7% in the chemotherapy cohort (6/29) had N3 disease. The substantial high disease burden may be associated with a higher risk of treatment failure with a lower pCR prior to upfront surgery, as well as an increased probability of disease recurrence following neoadjuvant treatment in the future.

A small number of patients in stage I (i.e., TNM stage cT1N0) were included in this retrospective descriptive study in order to reflect treatment decisions made in real-life settings in clinical multi-disciplinary meetings, i.e., allowing for those of very young age. Because each clinical scenario differs and treatment decisions vary on a case-to-case basis, their inclusion should not be interpreted as suggesting that the KN522 regimen is a standard for all patients with T1N0 disease.

Overall, 44% of patients were postmenopausal in the original trial [21], whereas 80% of patients in our study were postmenopausal. About 90% of patients in the original trial were <65 years old, with median age of 49 years [21], as opposed to the median age of 56 years in the pembrolizumab cohort in our study. This marks a huge contrast to conventional clinical trial setups, which typically favour younger and fitter patients. Denkert et al. established that tumour-infiltrating lymphocytes (TILs) serve as a strong predictor of pCR in TNBCs with younger age with higher baseline TIL density and superior pathological response [22]. Loi et al. showed that TNBC tumours in older, postmenopausal patients exhibit lower TIL level and lower pCR rate compared to those in younger patients [23]. Since older patients exhibit lower TIL levels, they tend to experience lower response rates to standard neoadjuvant treatment [22]. Older and frailer patients may experience more frequent dose modification, potential poorer treatment tolerance and an influence on the pCR rate.

In daily clinical practice, balancing treatment compliance with toxicity management remains a challenge in high-risk patient groups, especially those who are older and with higher nodal burden.

The treatment delivery findings provide practical information about the implementation of the KN522 regimen. The toxicity profile included both chemotherapy-related and immunotherapy-related adverse events. Grade 3 or higher chemotherapy-related adverse events were recorded in 50% of the KN522 cohort. Overall, 18 of 30 patients (60%) in the KN522 cohort required a certain degree of chemotherapy dose modification, similar to those in the UK cohort (52.7%) [11]. The overall side effect profile is consistent with other real-world studies and side effects were usually manageable.

Consistent with other real-world studies on a KN522 regimen in early-stage TNBC [13,14,23,24,25,26,27], our overall irAE profile (36.6%) was lower than that reported in the UK nationwide real-world cohort (60.6–68.8%) [11], but higher than that reported in the Korean real-world cohort (14.4%) [12]. Our Grade 3 or above irAE rate (13.3%) was similar to that from the nationwide UK cohort (19.6%) [11], but again higher than that in the Korean cohort (4.6%) [12]. Our pembrolizumab suspension rate (20%) was slightly higher than in the UK cohort (16.1%); all our patients who discontinued pembrolizumab continued with neoadjuvant chemotherapy alone. All irAE presented in our recruited patients were managed according to international recommendations [28]. This highlights that safe delivery requires not only oncological input but also rapid access to multi-disciplinary management with endocrinologist, gastroenterologist, dermatologist and physician input.

Interestingly, despite frequent chemotherapy dose adjustment, none of the patients in our KN522 cohort required early termination of neoadjuvant treatment as compared with 25.7% of patients in the UK nationwide RWD cohort [11]. Overall, 33.3% of our KN522 cohort patients who experienced irAE required steroid intervention, as compared with 38.9% in the UK nationwide RWD cohort [11]. This high chemotherapy modification rate reflects the vulnerability of older patients and the high N3 disease burden of our unselected patient population and may have contributed to the observed attenuation in pCR rate (43.3%) and early exploratory EFS events. These observations illustrate how real-world case demographics and treatment delivery may influence outcomes when a design trial regimen is implemented in clinical practice.

The observed exploratory EFS pattern may partly reflect the higher-risk clinical profile in the real-world setting. The unadjusted Cox estimate and log rank test are presented in a Supplementary Table for transparency and should not be interpreted as evidence of superior or inferior pembrolizumab efficacy. Only 10 EFS events occurred overall, resulting in substantial imprecision around the unadjusted hazard ratio. EFS also reflects the entire perioperative treatment pathway. Adjuvant pembrolizumab was administered to 76.7% of patients in the KN522 cohort, and adjuvant capecitabine was administered to 13.3% in the KN522 cohort and 65.5% in the dose-dense chemotherapy cohort. As the adjuvant treatment regimen varies in clinical practice, making it necessary to acknowledge that there are differences in baseline risk and chemotherapy backbone, the observed exploratory EFS findings should be considered hypothesis-generating and require confirmation in larger, multi-centre prospective studies with longer follow-up.

In the IMPassion030 trial, the addition of adjuvant atezolizumab added to chemotherapy did not improve invasive disease-free survival [29]. In the recent phase 3 NSABPB-59/GeparDouze trial, Loibl et al. reported that neoadjuvant atezolizumab increased pCR but did not statistically improve EFS outcome [30]. These findings suggest that treatment sequencing may influence the efficacy of immune checkpoint inhibition in early TNBC, with the administration of immunotherapy in the neoadjuvant phase potentially providing greater benefit than adjuvant immunotherapy alone. However, these findings differed from the KN522 trial in terms of choice of immunotherapy, treatment timing, chemotherapy backbone and study population. Nevertheless, these trials illustrate that improvements to pCR do not translate uniformly to improvements in EFS, and that chemotherapy intensity and treatment timing may contribute differently across treatment strategies.

Broader implementation of germline BRCA (gBRCA) testing in all TNBC patients warrants consideration, as approximately 10–15% of TNBC patients are gBRCA carriers [31]. Firstly, the adjuvant treatment arena following neoadjuvant treatment is evolving. The OlympiA trial demonstrated improvement in invasive disease-free survival with adjuvant Olaparib in gBRCA carriers with residual disease after neoadjuvant treatment [32]. Although no phase 3 trial has evaluated adjuvant pembrolizumab in combination with adjuvant Olaparib, treatment intensification in adjuvant settings represents a promising direction for reducing the risk of disease recurrence in high-risk TNBC patients. Secondly, a real-world study in a Latin American population reported a trend toward improved long-term outcomes and higher pCR in patients with BRCA mutations compared to a BRCA wide-type population [33].

### Limitations

Several limitations should be considered when interpreting these findings. The study was retrospective and conducted at a single centre, which limits generalisability and allows for unmeasured confounding between the treatment groups. The sample size was small, with 59 patients in total across the two cohorts, which reduced statistical power and produced wide confidence intervals. The small sample size reduced precision and limited the ability to detect clinically meaningful between-cohort differences. Moreover, patients in both cohorts received different post-adjuvant treatment pathways, which may contribute to the differences in survival. The survival analyses were also exploratory, as only ten event-free survival events occurred. The follow-up period was relatively short, and later exploratory Kaplan–Meier estimates were based on few patients at risk, overall hindering a comprehensive survival outcome analysis.

## 5. Conclusions

To our knowledge, this is among the first real-world studies describing the implementation of the KN522 regimen in a Hong Kong Chinese cohort that includes older patients who had substantial nodal burden. This study provides practical information on treatment delivery, toxicity, and early outcomes of the KEYNOTE-522 regimen in Chinese patients with early TNBC, as well as real-world evidence that neoadjuvant pembrolizumab-based therapy can be delivered in a Chinese TNBC population with higher risk factors. Side effects were found to be manageable with careful monitoring and early intervention. These findings support careful patient selection, proactive toxicity monitoring and efforts to maintain treatment intensity where clinically appropriate, which would be crucial to improve overall treatment outcomes. Further larger multi-centre evaluations with longer follow-up periods are warranted.

## Figures and Tables

**Table 1:** Baseline demographic and clinical characteristics by treatment cohort.

Characteristic	Category/Statistic	Overall (*N* = 59)	KN522 Cohort (*n* = 30)	Chemotherapy Cohort (*n* = 29)	*p*
Age, years	Mean ± SD	57.2 ± 9.7	57.9 ± 10.5	56.4 ± 9.0	0.704
	Median [IQR]	56.0 (51.0, 64.0)	56.0 (51.0, 65.5)	57.0 (51.0, 62.0)	
Menopausal status	Premenopausal	15 (25.4%)	6 (20.0%)	9 (31.0%)	0.330
	Postmenopausal	44 (74.6%)	24 (80.0%)	20 (69.0%)	
ECOG performance status	ECOG 0	5 (8.5%)	2 (6.7%)	3 (10.3%)	0.508
	ECOG 1	53 (89.8%)	28 (93.3%)	25 (86.2%)	
	ECOG 2	1 (1.7%)	0 (0.0%)	1 (3.4%)	
Clinical T stage	Tis	1 (1.7%)	0 (0.0%)	1 (3.4%)	0.366
	T1	4 (6.8%)	3 (10.0%)	1 (3.4%)	
	T2	44 (74.6%)	24 (80.0%)	20 (69.0%)	
	T3	5 (8.5%)	1 (3.3%)	4 (13.8%)	
	T4	5 (8.5%)	2 (6.7%)	3 (10.3%)	
Clinical N stage	N0	24 (40.7%)	14 (46.7%)	10 (34.5%)	0.121
	N1	19 (32.2%)	6 (20.0%)	13 (44.8%)	
	N2	0 (0%)	0 (0%)	0 (0%)	
	N3	16 (27.1%)	10 (33.3%)	6 (20.7%)	
Node-positive disease	Yes	35 (59.3%)	16 (53.3%)	19 (65.5%)	0.341
	No	24 (40.7%)	14 (46.7%)	10 (34.5%)	
Overall clinical stage	Stage I	3 (5.1%)	2 (6.7%)	1 (3.4%)	1.00
	Stage II	34 (57.6%)	17 (56.7%)	17 (58.6%)	
	Stage III	22 (37.3%)	11 (36.7%)	11 (37.9%)	
Any comorbidity	No	34 (57.6%)	19 (63.3%)	15 (51.7%)	0.367
	Yes	25 (42.4%)	11 (36.7%)	14 (48.3%)	

Note: Exploratory *p*-values are presented for descriptive transparency only and should not be interpreted as evidence of between-cohort comparability or treatment effects.

**Table 2:** Pathological complete response by treatment cohort with external benchmark context.

Analysis Component	Chemotherapy Cohort (*n* = 29)	KN522 Cohort (*n* = 30)	Estimate/Statistic
Current cohort outcome			
pCR, n/N (%)	9/29 (31.0%)	13/30 (43.3%)	
non-pCR, n/N (%)	20/29 (69.0%)	17/30 (56.7%)	
pCR rate, exact 95% CI	15.3% to 50.8%	25.5% to 62.6%	
External benchmark context			
KEYNOTE-522 pembrolizumab + chemotherapy cohort			64.8%
ESMO real-world pembrolizumab + chemotherapy cohort			58.0%

Note. pCR = pathological complete response; CI = confidence interval; pp = percentage points. In the current study, pCR was defined as no residual invasive disease in the breast and sampled nodes at surgery, corresponding to ypT0/Tis ypN0 where pathology information was available. Exact binomial 95% confidence intervals are shown separately by treatment cohort. External benchmark rates from KEYNOTE-522 and the ESMO real-world cohort are presented for descriptive context only and were not statistically compared with the current cohort.

**Table 3:** Safety and treatment delivery outcomes by treatment cohort.

Outcome	Overall (*N* = 59)	Chemotherapy Cohort (*n* = 29)	KN522 Cohort (*n* = 30)	Exploratory *p*-Value
Toxicity				
Any-grade chemotherapy-related adverse event	54/59 (91.5%)	25/29 (86.2%)	29/30 (96.7%)	0.195
Grade ≥3 chemotherapy-related adverse event	21/59 (35.6%)	6/29 (20.7%)	15/30 (50.0%)	0.019
Treatment modification				
Dose modification	34/59 (57.6%)	16/29 (55.2%)	18/30 (60.0%)	0.708
Lowest chemotherapy dose %, median [IQR]	80.0 (75, 100)	90.0 (80, 100)	80.0 (75, 100)	0.513
Lowest chemotherapy dose %, mean ± SD	86.7 ± 12.8	87.9 ± 12.3	85.5 ± 13.3	
Pembrolizumab suspension			6/30 (20.0%)	
Immune-related intervention			10/30 (33.3%)	
Early termination of neoadjuvant treatment	2/59 (3.4%)	2/29 (6.9%)	0/30 (0.0%)	0.237
Surgery and postoperative treatment				
Definitive surgery completed	59/59 (100.0%)	29/29 (100.0%)	30/30 (100.0%)	-
Postoperative radiotherapy	46/59 (78.0%)	22/29 (75.9%)	24/30 (80.0%)	0.701
Adjuvant treatment category				<0.001
Capecitabine/Xeloda	23/59 (39.0%)	19/29 (65.5%)	4/30 (13.3%)	
Pembrolizumab	23/59 (39.0%)	0/29 (0.0%)	23/30 (76.7%)	
				
IO discontinued	1/59 (1.7%)	0/29 (0.0%)	1/30 (3.3%)	
Hormonal therapy	1/59 (1.7%)	1/29 (3.4%)	0/30 (0.0%)	
Progressed after surgery	1/59 (1.7%)	0/29 (0.0%)	1/30 (3.3%)	

Note. Values are presented as *n*/*N*(%). *p*-values represent exploratory comparisons between the chemotherapy and KN522 cohorts. Cohort-specific percentages are descriptive. *p*-values represent exploratory cross-cohort comparisons and are provided for transparency; they should not be interpreted as causal estimates of treatment effects. The cohorts differed in chemotherapy backbone, pembrolizumab exposure, treatment selection, and postoperative treatment. AE = Adverse event; CI = confidence interval; IQR = interquartile range; irAE = immune-related adverse event; IO = immunotherapy; RT = radiotherapy.

**Table 4:** Descriptive summary of event-free survival by treatment cohort.

Group	N	EFS Events	No EFS Event	Event Dates Available	Median Time to Event, Months
Overall	59	10/59 (16.9%)	49/59 (83.1%)	10	21.7
Chemotherapy cohort	29	3/29 (10.3%)	26/29 (89.7%)	3	15.2
KN522 cohort	30	7/30 (23.3%)	23/30 (76.7%)	7	23.2

Note. EFS = event-free survival. Event timing was calculated from treatment start to the first recorded EFS event. Patients without an EFS event were censored at the administrative data cut-off date of 12 July 2026 for the exploratory Kaplan–Meier analysis.

**Table 5:** Descriptive event-free survival outcomes by treatment cohort.

Measure	Overall (*N* = 59)	Chemotherapy (*n* = 29)	KN522 (*n* = 30)
EFS events, n/N (%)	10/59 (16.9%)	3/29 (10.3%)	7/30 (23.3%)
Censored, no EFS event	49/59 (83.1%)	26/29 (89.7%)	23/30 (76.7%)
Median follow-up, months (95% CI)	28.2 (24.3, 34.4)	31.5 (27.5, 39.0)	25.7 (20.2, 36.7)
12-month EFS, % (95% CI)	96.6 (92.1, 100.0)	96.6 (90.1, 100.0)	96.7 (90.5, 100.0)
24-month EFS, % (95% CI)	85.2 (75.5, 96.2)	88.3 (76.7, 100.0)	80.6 (64.7, 100.0)

## Data Availability

All data generated or analysed during the present study are available from the corresponding author on reasonable request.

## Supplementary Materials

The following supporting information can be downloaded at https://www.mdpi.com/article/10.3390/cancers18203339/s1: Table S1: Pathological Complete Response by Treatment Cohort with External Benchmark Context; Table S2: Immune-Related Adverse Events by Type and Grade; Table S3: Univariable and Multivariable Exploratory Logistic regression Analyses for Pathological Complete Response; Table S4: Descriptive Event Free Survival Outcomes by Treatment Cohort. Figure S1: Detailed Adverse Event Profile by Type and Severity. Note. AE = adverse event; Figure S2: Immune-Related Adverse Event Profile in the KN522 Cohort; Figure S3: Kaplan–Meier Curves for Event-Free Survival by Treatment Cohort.
