# Supplementary material for: Real-World Feasibility, Toxicity, and Early Outcomes of Neoadjuvant Pembrolizumab-Based Systemic Therapy in Early Triple-Negative Breast Cancer: A Single-Centre Chinese Cohort from Hong Kong
Source: Cancers (Basel). 2026 Oct 16;18(20):3339. doi: 10.3390/cancers18203339 (PMC13702728; doi:10.3390/cancers18203339)
Supplement: Supplementary file 1 [file cancers-18-03339-s001.zip › cancers-4608715-supplementary.pdf]

cancers

MDPI

Supplementary tables

Table S1. Pathological Complete Response by Treatment Cohort with External Benchmark Context.

Analysis component	Chemotherapy cohort *(n = 29)*	KN522 cohort *(n = 30)*	Estimate / statistic
Current cohort outcome			
pCR, n/N (%)	9/29 (31.0%)	13/30 (43.3%)	
non-pCR, n/N (%)	20/29 (69.0%)	17/30 (56.7%)	
pCR rate, exact 95% CI	15.3% to 50.8%	25.5% to 62.6%	
Between-cohort comparison			
Absolute risk difference			12.3 pp (-12.2 to 36.8)
Odds ratio for pCR			1.68 (0.52 to 5.70)
Fisher's exact test			$p = 0.422$
Pearson chi-square sensitivity test			$p = 0.329$
External benchmark context			
KEYNOTE-522 pembrolizumab + chemotherapy cohort			64.8%
ESMO real-world pembrolizumab + chemotherapy cohort			58.0%

Note. pCR = pathological complete response; CI = confidence interval; pp = percentage points. In the current study, pCR was defined as no residual invasive disease in the breast and sampled nodes at surgery, corresponding to ypT0/Tis ypN0 where pathology information was available. Exact binomial 95% confidence intervals are shown separately by treatment cohort. The absolute risk difference was calculated as the KN522 cohort minus the chemotherapy cohort. The odds ratio compares the odds of pCR in the KN522 cohort with the chemotherapy cohort as the reference group. Fisher's exact test was used as the primary between-cohort comparison because this was a small retrospective cohort with a 2 × 2 outcome table; Pearson chi-square was retained as a sensitivity analysis. External benchmark rates from KEYNOTE-522 and the ESMO real-world cohort are presented for descriptive context only and were not statistically compared with the current cohort.

Table S2. Immune-Related Adverse Events by Type and Grade.

irAE type	Overall any grade	Chemotherapy any grade	KN522 any grade	Overall grade ≥3	Chemotherapy grade ≥3	KN522 grade ≥3	Max grade KN522
Adrenal insufficiency	1/59 (1.7%)	0/29 (0.0%)	1/30 (3.3%)	0/59 (0.0%)	0/29 (0.0%)	0/30 (0.0%)	2
Gastritis	0/59 (0.0%)	0/29 (0.0%)	0/30 (0.0%)	0/59 (0.0%)	0/29 (0.0%)	0/30 (0.0%)	0
Hepatitis	3/59 (5.1%)	0/29 (0.0%)	3/30 (10.0%)	3/59 (5.1%)	0/29 (0.0%)	3/30 (10.0%)	4
Hyperthyroidism	1/59 (1.7%)	0/29 (0.0%)	1/30 (3.3%)	0/59 (0.0%)	0/29 (0.0%)	0/30 (0.0%)	1
Hypophysitis	0/59 (0.0%)	0/29 (0.0%)	0/30 (0.0%)	0/59 (0.0%)	0/29 (0.0%)	0/30 (0.0%)	0

irAE type	Overall any grade	Chemotherapy any grade	KN522 any grade	Overall grade ≥3	Chemotherapy grade ≥3	KN522 grade ≥3	Max grade KN522
Hypothyroidism	3/59 (5.1%)	0/29 (0.0%)	3/30 (10.0%)	0/59 (0.0%)	0/29 (0.0%)	0/30 (0.0%)	2
Pneumonitis	1/59 (1.7%)	0/29 (0.0%)	1/30 (3.3%)	0/59 (0.0%)	0/29 (0.0%)	0/30 (0.0%)	2
Severe skin reaction	2/59 (3.4%)	0/29 (0.0%)	2/30 (6.7%)	1/59 (1.7%)	0/29 (0.0%)	1/30 (3.3%)	3
Thyroiditis	0/59 (0.0%)	0/29 (0.0%)	0/30 (0.0%)	0/59 (0.0%)	0/29 (0.0%)	0/30 (0.0%)	0

Note. Values are n/N (%). Any-grade irAE was defined as grade ≥1; grade ≥3 irAE was defined as grade ≥3. The maximum grade columns describe the highest recorded grade overall and in the KN522 cohort. irAE = immune-related adverse event.

Table S3. Univariable and Multivariable Exploratory Logistic-regression Analyses for Pathological Complete Response.

Predictor	Unadjusted OR (95% CI); p	Model 1: cohort + overall clinical stage OR (95% CI); p	Model 2: cohort + node-positive disease OR (95% CI); p
Treatment cohort			
KN522 vs chemotherapy	1.70 (0.58–4.94) *p = 0.330*	1.74 (0.59–5.17) *p = 0.317*	1.60 (0.54–4.72) *p = 0.395*
Overall clinical stage			
Stage II vs Stage I	1.58 (0.13–19.12) *p = 0.720*	1.75 (0.14–21.71) *p = 0.665*	
Stage III vs Stage I	0.75 (0.06–9.87) *p = 0.827*	0.82 (0.06–11.02) *p = 0.880*	
Node-positive disease			
Node-positive vs node-negative	0.54 (0.19–1.59) *p = 0.263*	—	0.57 (0.19–1.69) *p = 0.311*
Age			
Per 10-year increase	0.55 (0.30–1.01) *p = 0.052*	—	—
Menopausal status			
Premenopausal vs postmenopausal	1.69 (0.51–5.56) *p = 0.387*	—	—
ECOG performance status			
ECOG 1 vs 0	0.37 (0.06–2.43) *p = 0.302*	—	—
ECOG 2 vs 0	*NE*	—	—
Clinical T stage			
T2 vs T1	2.50 (0.24–25.95) *p = 0.443*	—	—
T3 vs T1	*NE*	—	—
T4 vs T1	0.75 (0.03–17.51) *p = 0.858*	—	—

Predictor	Unadjusted OR (95% CI); p	Model 1: cohort + overall clinical stage OR (95% CI); p	Model 2: cohort + node-positive disease OR (95% CI); p
Tis vs T1	*NE*	—	—
Clinical N stage			
N1 vs N0	0.42 (0.12–1.55)<br>*p = 0.193*	—	—
N3 vs N0	0.71 (0.19–2.58)<br>*p = 0.602*	—	—
Any comorbidity			
Present vs absent	0.36 (0.11–1.11)<br>*p = 0.075*	—	—

Note. Outcome variable was pathological complete response. Values are odds ratios with 95% confidence intervals, followed by p values. OR = odds ratio; CI = confidence interval; ECOG = Eastern Cooperative Oncology Group; NE = not estimable because of sparse separation. Unadjusted estimates were derived from separate univariable logistic regression models using Wald test. Model 1 included treatment cohort and overall clinical stage. Model 2 included treatment cohort and node-positive disease. Reference categories were chemotherapy cohort, postmenopausal status, ECOG 0, T1, N0, node-negative disease, Stage I, and absence of comorbidity. Age was modelled as a continuous variable, with the OR representing the change in odds of pCR per 10-year increase. Models were exploratory because of the limited sample size and number of pCR events.

Table S4. Descriptive Event Free Survival Outcomes by Treatment Cohort.

Measure	Overall<br>*(N = 59)*	Chemotherapy<br>*(n = 29)*	KN522<br>*(n = 30)*	*p*
EFS events, n/N (%)	10/59 (16.9%)	3/29 (10.3%)	7/30 (23.3%)	
Censored, no EFS event	49/59 (83.1%)	26/29 (89.7%)	23/30 (76.7%)	
Median follow-up, months (95% CI)	28.2 (24.3, 34.4)	31.5 (27.5, 39.0)	25.7 (20.2, 36.7)	
Median EFS, months	Not reached	Not reached	Not reached	
12-month EFS, % (95% CI)	96.6 (92.1, 100.0)	96.6 (90.1, 100.0)	96.7 (90.5, 100.0)	
24-month EFS, % (95% CI)	85.2 (75.5, 96.2)	88.3 (76.7, 100.0)	80.6 (64.7, 100.0)	
Log-rank test		Reference	KN522 vs chemotherapy	0.108
Unadjusted Cox HR (95% CI)		Reference	2.91 (0.75, 11.37)	0.124

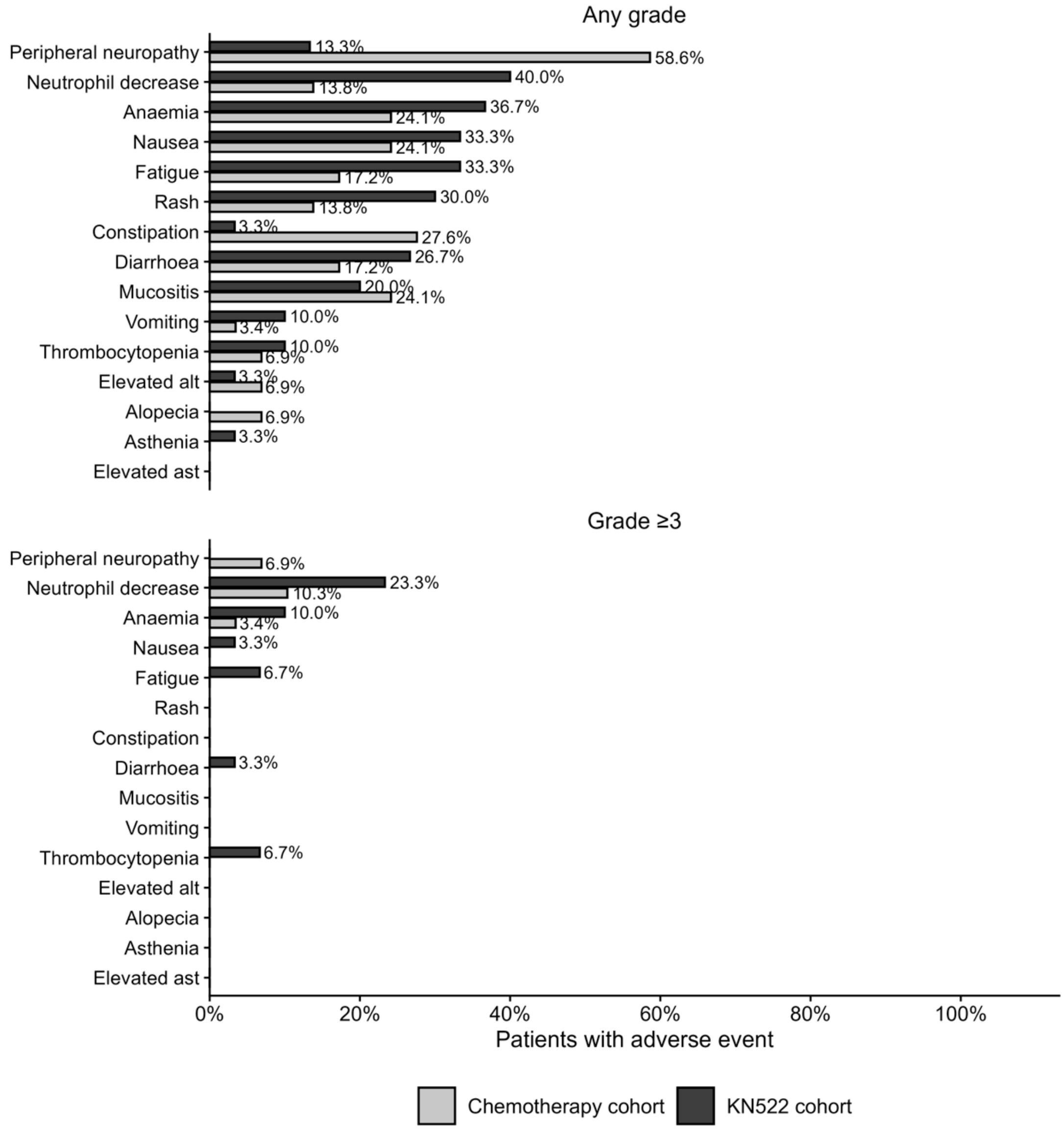

Figure S1. Detailed Adverse-Event Profile by Type and Severity. Note. AE = adverse event. Any-grade AE was defined as grade ≥1; grade ≥3 AE was defined as grade ≥3.

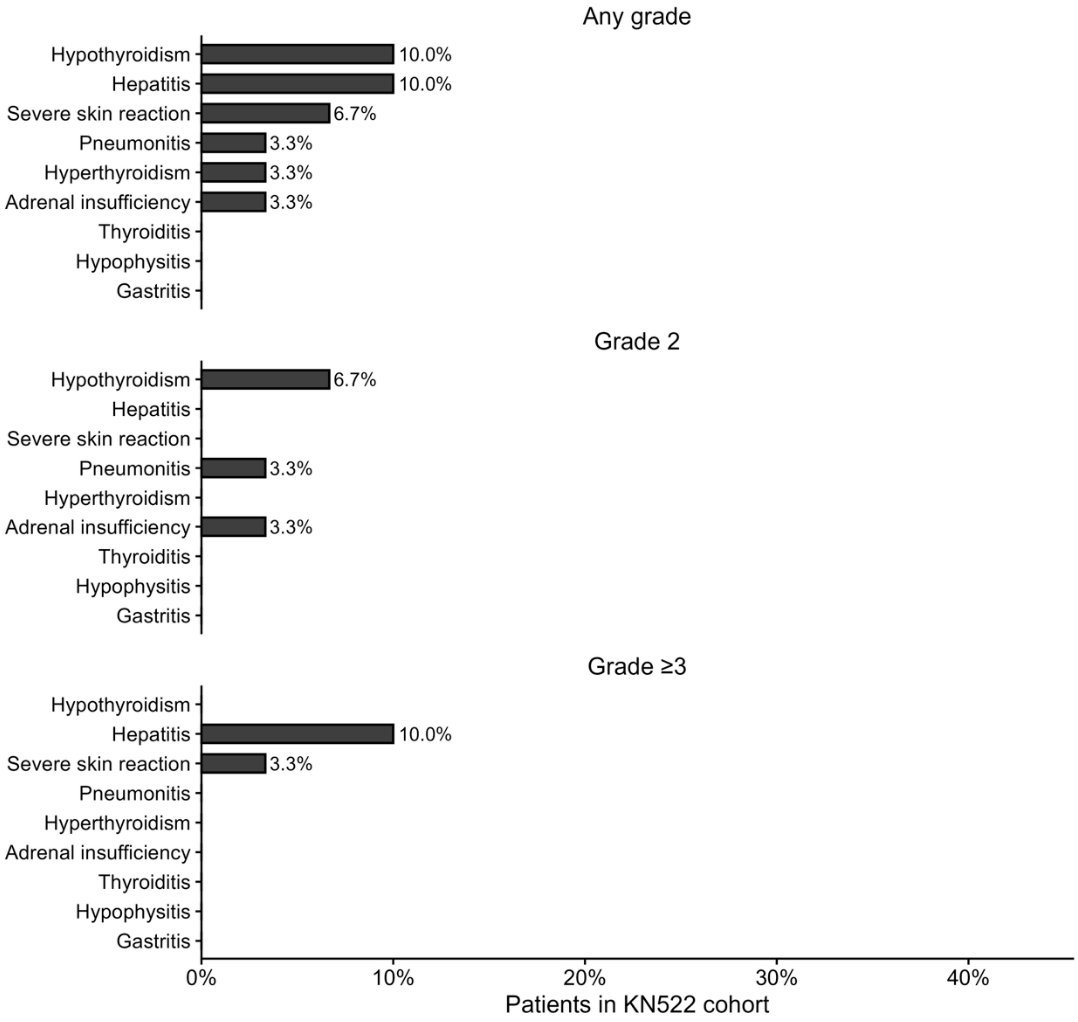

Figure S2. Immune-Related Adverse-Event Profile in the KN522 Cohort. Note. irAE = immune-related adverse event. The denominator is the KN522 cohort. Pembrolizumab-specific outcomes should be interpreted within the KN522 group.

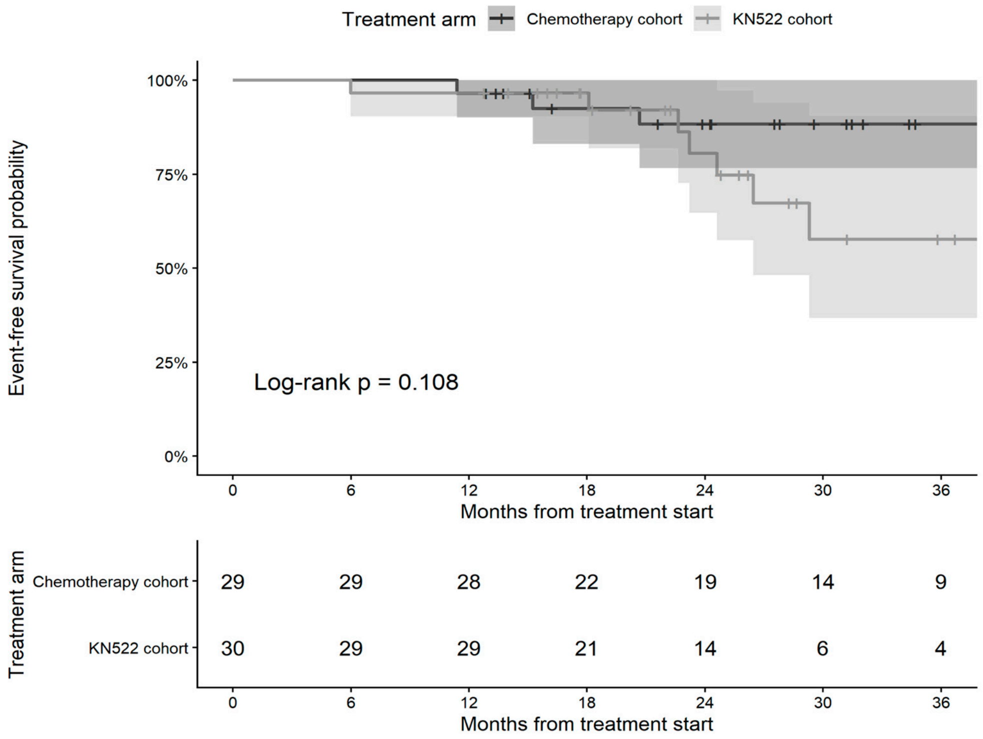

Figure S3. Kaplan-Meier Curves for Event-Free Survival by Treatment Cohort.
